# Antibiotic and Antiinflammatory Therapy Transiently Reduces Inflammation and Hypercoagulation in Acutely SIV-Infected Pigtailed Macaques

**DOI:** 10.1371/journal.ppat.1005384

**Published:** 2016-01-14

**Authors:** Ivona Pandrea, Cuiling Xu, Jennifer L. Stock, Daniel N. Frank, Dongzhu Ma, Benjamin B. Policicchio, Tianyu He, Jan Kristoff, Elaine Cornell, George S. Haret-Richter, Anita Trichel, Ruy M. Ribeiro, Russell Tracy, Cara Wilson, Alan L. Landay, Cristian Apetrei

**Affiliations:** 1 Center for Vaccine Research, University of Pittsburgh, Pittsburgh, Pennsylvania, United States of America; 2 Department of Pathology, School of Medicine, University of Pittsburgh, Pittsburgh, Penssylvania, United States of America; 3 Department of Medicine, University of Colorado, Aurora, Colorado, United States of America; 4 Department of Microbiology and Molecular Genetics, School of Medicine, University of Pittsburgh, Pittsburgh, Penssylvania, United States of America; 5 Department of Pathology and Laboratory Medicine, University of Vermont, Burlington, Vermont, United States of America; 6 Division of Laboratory Animal Resources, School of Medicine, University of Pittsburgh, Pittsburgh, Pennsylvania, United States of America; 7 Theoretical Biology and Biophysics Group, Los Alamos National Laboratory, Los Alamos, New Mexico, United States of America; 8 Department of Immunology and Microbiology, Rush University Medical Center, Chicago, Illinois, United States of America; Emory University, UNITED STATES

## Abstract

Increased chronic immune activation and inflammation are hallmarks of HIV/SIV infection and are highly correlated with progression to AIDS and development of non-AIDS comorbidities, such as hypercoagulability and cardiovascular disease. Intestinal dysfunction resulting in microbial translocation has been proposed as a lead cause of systemic immune activation and hypercoagulability in HIV/SIV infection. Our goal was to assess the biological and clinical impact of a therapeutic strategy designed to reduce microbial translocation through reduction of the microbial content of the intestine (Rifaximin-RFX) and of gut inflammation (Sulfasalazine-SFZ). RFX is an intraluminal antibiotic that was successfully used in patients with hepatic encephalopathy. SFZ is an antiinflammatory drug successfully used in patients with mild to moderate inflammatory bowel disease. Both these clinical conditions are associated with increased microbial translocation, similar to HIV-infected patients. Treatment was administered for 90 days to five acutely SIV-infected pigtailed macaques (PTMs) starting at the time of infection; seven untreated SIVsab-infected PTMs were used as controls. RFX+SFZ were also administered for 90 days to three chronically SIVsab-infected PTMs. RFX+SFZ administration during acute SIVsab infection of PTMs resulted in: significantly lower microbial translocation, lower systemic immune activation, lower viral replication, better preservation of mucosal CD4^+^ T cells and significantly lower levels of hypercoagulation biomarkers. This effect was clear during the first 40 days of treatment and was lost during the last stages of treatment. Administration of RFX+SFZ to chronically SIVsab–infected PTMs had no discernible effect on infection. Our data thus indicate that early RFX+SFZ administration transiently improves the natural history of acute and postacute SIV infection, but has no effect during chronic infection.

## Author Summary

We report that administration of the intraluminal antibiotic Rifaximin and the gut-focused anti-inflammatory drug Sulfasalazine to acutely SIV-infected pigtailed macaques is associated with a transient disruption of the vicious circle of inflammation-microbial translocation-immune activation which is pathognomonic to pathogenic HIV/SIV infection and drives HIV disease progression and non-AIDS comorbidities in HIV-infected patients. This therapeutic approach resulted in transient lower microbial translocation, lower systemic immune activation, lower viral replication, better preservation of mucosal CD4^+^ T cells and lower levels of hypercoagulation biomarkers throughout acute SIV infection. Our results thus support the use of therapeutic approaches to reduce microbial translocation, improve the clinical outcome of HIV-infected patients receiving antiretroviral therapy and prevent non-AIDS comorbidities. Our results also reinforce the importance of early therapeutic management of HIV infection.

## Introduction

The current paradigm of HIV/SIV pathogenesis is that chronic systemic immune activation is a major determinant of progression to AIDS, independent of the levels of chronic viral replication or CD4^+^ T cell depletion [1]. Several lines of evidence support this paradigm. In both HIV-infected patients and SIV-infected macaques on antiretroviral therapy (ART), poor prognosis and high incidence of non-AIDS comorbidities are associated with residual increased levels of immune activation, and not with viral replication, which is controlled with antiretroviral (ARV) drugs [2–4]. Additionally, comparative studies between progressive and nonprogressive animal models of HIV infection [5–7] revealed that the lack of disease progression in natural hosts of SIVs is due to their ability to control immune activation rather than contain virus replication during chronic infection [8–15]. In stark contrast, pathogenic SIV infection of macaques is associated with high levels of immune activation and inflammation, as illustrated by increases in both adaptive and innate immune effectors [16–18].

The consequences of persistent immune activation during chronic HIV/SIV infection are plentiful but not completely elucidated [19]. In HIV-infected patients and SIV-infected macaques receiving ART, residual increased levels of systemic and tissue T cell immune activation strongly correlate with incomplete immune restoration [20], non-AIDS comorbidities and premature aging [21]. Therefore, it is critical to identify therapies that resolve immune activation, with the goal to improve the response to ART, increase the quality of life and ultimately prolong survival in HIV-infected patients [21].

The causes of the persistent immune activation in the clinical setting of controlled virus replication in patients and macaques on ART are not yet fully elucidated, which is a major obstacle to the achievement of complete therapeutic success. Systemic immune activation is correlated with increased levels of systemic circulating microbial products translocated from the intestinal lumen, as a consequence of the severe mucosal damage occurring during HIV infection [22–25]. These microbial products translocate relatively early during HIV/SIV infection and overstimulate numerous innate and adaptive immune effectors, resulting in excessive inflammation [22–25]. Strong support for a significant role of microbial translocation in triggering chronic immune activation in HIV-infected patients also comes from studies of SIV-infected NHPs. In macaques, progression to AIDS is correlated with increased levels of microbial translocation and circulating microbial products [24], while the lack of disease progression in the natural African NHP hosts of SIVs is associated with maintenance of the mucosal barrier and no significant change in microbial translocation [11,26–28]. Furthermore, experimental modeling of microbial translocation in natural hosts resulted in increased levels of systemic immune activation and CD4^+^ T cell depletion [29,30]. Altogether, these NHP studies point to microbial translocation as a key determinant in driving excessive chronic immune activation during HIV/SIV infection. Early HIV/SIV-induced intestinal damage results in increased gut inflammation, which in turn fuels virus replication that deepens the intestinal dysfunction favoring increased microbial translocation, further increasing systemic immune activation, non-AIDS comorbidities and ultimately progression to AIDS [1].

With the goal of breaking this vicious cycle of AIDS pathogenesis, we designed a therapeutic strategy based on a combination of a luminal antibiotic (Rifaximin-RFX) and a gut-targeting antiinflammatory drug (Sulfasalazine-SFZ). RFX is an intraluminal antibiotic with broad activity against Gram^neg^, Gram^pos^, *Mycobacteria* and anaerobes that is used in patients with hepatic encephalopathy, and can reduce overall intestinal microbial burden [31]. SFZ is used in patients with inflammatory bowel disease and can reduce mucosal inflammation [32]. Both clinical categories are associated with elevated microbial translocation levels, similar to those observed in HIV-infected patients [22,25]. Our therapeutic approach was thus designed to reduce both the levels of intestinal inflammation and microbial burden in the intestine and ultimately reduce microbial translocation-induced systemic immune activation in SIVsab-infected PTMs. We report that early administration of RFX+SFZ to acutely SIV-infected PTMs significantly reduced levels of microbial translocation and systemic immune activation, limited mucosal CD4^+^ T cell loss, and reduced the levels of viral replication. We also report that administration of both drugs to ART-naïve chronically SIVsab-infected PTMs failed to modify these key parameters of infection.

## Results

### Study design

Fifteen PTMs were challenged intravenously with plasma equivalent to 300 TCID50 of SIVsabBH66. At the time of virus inoculation, five PTMs (treated group; red in graphs) received oral therapy with RFX (400 mg/day) and SFZ (dosed at 75 mg/kg for the first month of treatment, then adjusted to 25 mg/kg for two months). These doses were in the range of those administered to human subjects [31–33]. The rationale for the adjustment of the SFZ dose at the end of the acute infection is that we reasoned that a higher dose of SFZ would better control the higher levels of inflammation characteristic to acute SIV infection, while at the end of acute infection, with the partial resolution of inflammation, it would be appropriate to bring the SFZ dose to a maintenance dose similar to that used in patients with inflammatory bowel disease. Total duration of therapy was 3 months. Seven PTMs were used as untreated SIVsab-infected controls (control group; black in graphs) in which infection followed its natural course.

We chose to initiate the RFX+SFZ treatment during the acute stage of SIV infection, despite the majority of HIV-infected patients receiving ART and presenting with residual immune activation are in more advanced stages of chronic infection, because the mucosal barrier damage inflicted by HIV/SIV occurs during the first few weeks of infection [23,34]. This early therapeutic approach thus permitted us to assess the efficacy of the RFX+SFZ treatment by measuring the major parameters of SIV infection before and after mucosal injury, without any interference of ART. To this end, we collected peripheral blood, duodenal and lymph node (LN) biopsies at multiple critical time points of infection, as shown in S1 Fig.

Due to the relatively small size of the groups and to variations in the different parameters between different animals, we used baseline levels of the tested parameters (average of three different time points) to normalize the results as fold increase from the baseline, except for viral load and peripheral CD4^+^ T cells. Thus, throughout the paper, graphs illustrate the arithmetic mean of the fold increase from baseline in both treated (in red) and control groups (in black). Detailed results are presented as supplementary information (S2–S6 Figs). We assessed treatment efficacy using mixed-effects models in two distinct periods: the acute phase, between 1 and 21 days postinfection (dpi), and the chronic stage of infection, >40 days post infection (see Methods for details).

### Rifaximin and sulfasalazine treatment significantly reduces the levels of microbial translocation in acutely SIVsab-infected PTMs

The ultimate goal of the administration of RFX+SFZ was to provide proof of concept data showing that lowering microbial translocation and impacting systemic inflammation and immune activation breaks the vicious circle of SIV pathogenesis.

Therefore, rather than assessing the independent effects of each drug, we first assessed the impact of the RFX+SFZ therapy, by comparing levels of circulating microbial products between treated and untreated PTMs. We used two different methods to assess the impact of RFX+SFZ therapy on systemic levels of microbial translocation.

**Fig 1:**
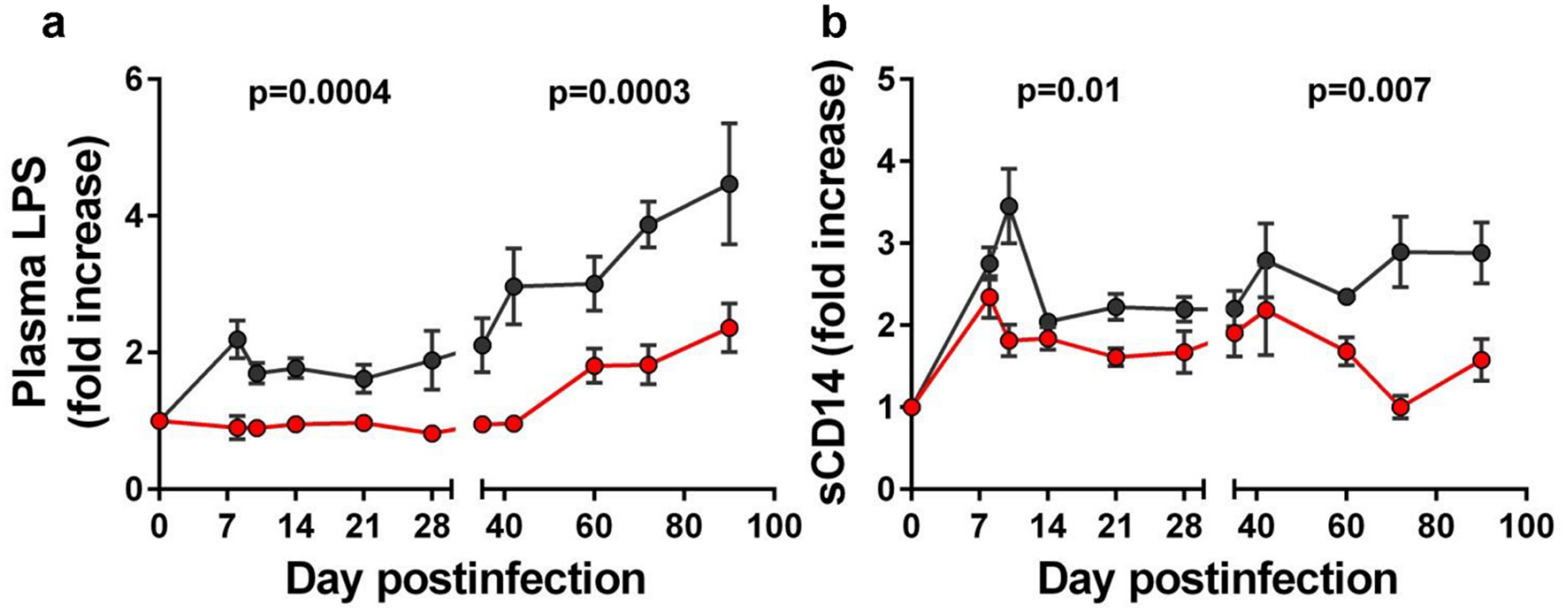
Rifaximin (RFX) + sulfasalazine (SFZ) treatment reduces microbial translocation during early SIVsab infection of pigtailed macaques (PTMs). (a) Comparison between plasma LPS levels in SIVsab-infected PTMs receiving RFX+SFZ (red) and untreated controls (black). (b) Comparison between plasma sCD14 levels in SIVsab-infected PTMs receiving RFX+SFZ (red) and untreated controls (black). The average values for each group and standard error of means are shown.

**Fig 2:**
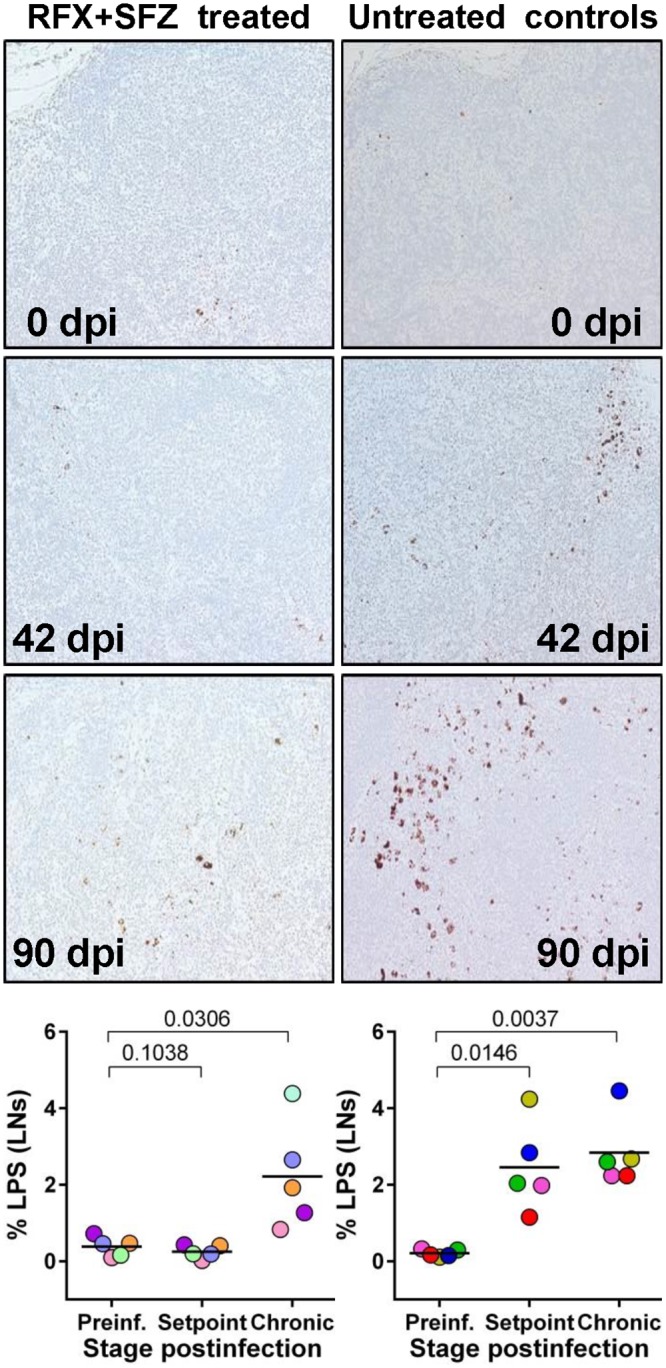
Comparison between the levels of microbial translocation in axillary lymph nodes (LNs) of SIVsab-infected PTMs receiving RFX+SFZ and untreated controls. Representative images (50x) of the LNs stained for LPS-core antigen (brown) collected prior to infection, at the set-point and at the end of treatment. Note the extensive accumulation of microbial products within the macrophages located around the subcapsular and medullary sinuses, and in the paracortical parenchyma of the lymphatic tissues in untreated controls, and moderate increase in LPS levels in LNs of PTMs treated with RFX+SFZ. Quantification of the percent area of the LN positive for LPS. P values are based on the Mann–Whitney test. Graphs show group means at critical times of SIV infection, with individual animal data points shown.

First, we measured LPS levels in plasma and tissues (Figs 1, 2 and S2). LPS levels in the bloodstream were shown in numerous studies to be an excellent surrogate of the breakdown of the gut-mucosal immune barrier in HIV/SIV infected humans and NHPs [24,34,35]. Using this method, we determined that administration of RFX+SFZ significantly reduced the levels of microbial products translocated into the bloodstream of treated PTMs compared to untreated controls throughout the follow-up (Fig 1a) (p = 0.0004 for the difference in the acute phase and p = 0.0003 for the early chronic stage of infection). However, LPS levels increased in treated PTMS during the early chronic stage of infection, indicating a loss of treatment efficacy.

Similar to LPS testing, the sCD14 assay showed that administration of RFX+SFZ significantly lowered the levels of macrophage activation throughout the follow-up (Figs 1b and S2). sCD14 levels were significantly lower in treated *vs*. untreated PTMs during acute infection (p = 0.01) and also in early chronic infection (p = 0.007) (Fig 1b).

Since plasma LPS levels are a reflection of both alterations in intestinal barrier integrity and of LPS clearance by the liver [22,25], we next assessed the levels of microbial translocation in SIVsab-infected PTMs using immunohistochemical staining for LPS [16]performed on peripheral LN samples collected at critical time points after SIVsab infection from treated and untreated PTMs. IHC revealed that the number of LPS positive macrophages was significantly lower in LNs of treated animals during the first 42 dpi. However, like for the plasma LPS levels, by the end of treatment we could detect a significant increase in the LPS levels in peripheral LNs in PTMs receiving RFX+SFZ therapy (Fig 2).

Altogether, these results demonstrate that administration of both an antibiotic and an antiinflammatory drug targeting both intraluminal microbial burden and local luminal inflammation transiently alleviates the levels of microbial translocation in acutely SIV-infected NHPs.

### Antibiotic and antiinflammatory therapy reduces the levels of T cell immune activation during acute SIVsab infection of PTMs

**Fig 3:**
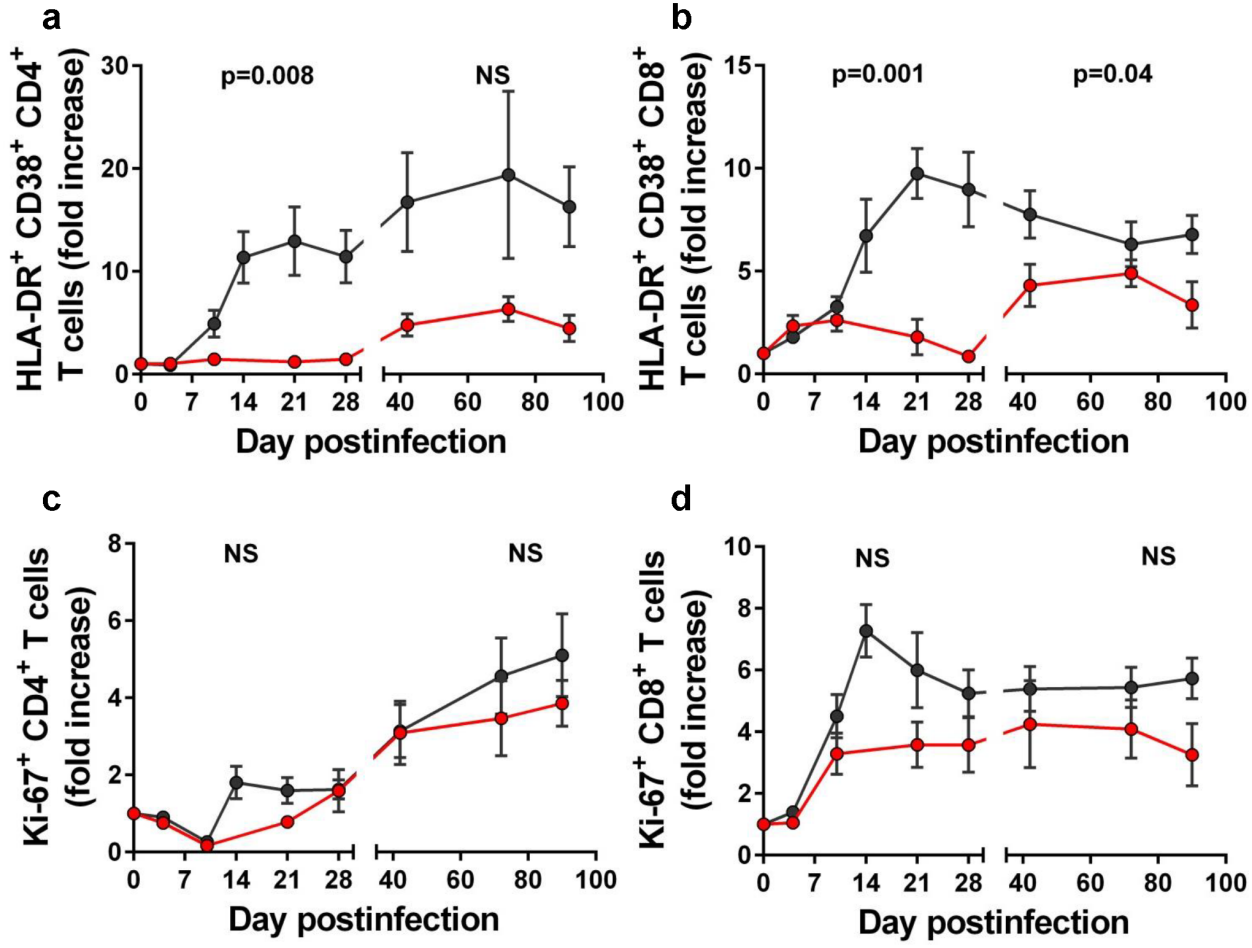
Rifaximin (RFX) + sulfasalazine (SFZ) therapy impacts T cell immune activation during acute and early chronic SIVsab infection of pigtailed macaques (PTMs). Significant differences were observed between SIVsab-infected PTMs receiving RFX+SFZ (red) and untreated controls (black) with regard to CD38 and HLA-DR expression by CD4^+^ T cells (a) and CD8^+^ T cells (b). Conversely, Ki-67 expression by CD4^+^ T cells (c) and CD8^+^ T cells (d) was not statistically different between the two groups. Shown are the average values for each group and standard error of means.

We next assessed the impact of RFX+SFZ treatment on systemic levels of T cell immune activation by comparing the expression of HLA-DR and CD38 on CD4^+^ and CD8^+^ T cells between treated and nontreated SIVsab-infected PTMs. HLA-DR and CD38 expression on both CD4^+^ T and CD8^+^ T cells was lower in the PTMs receiving the RFX+SFZ therapy compared to untreated controls (Figs 3a, 3b and S3). The differences between the two groups were significant throughout the acute infection for both CD4^+^ (p = 0.008), and in CD8^+^ T cells (p = 0.001) (Fig 3a and 3b). During chronic infection, the differences in HLA-DR expression were only significant for CD8^+^ T cells (p = 0.04) (Fig 3b), due to large variability in the CD4^+^ T cells of the control animals (Figs 3a and S3).

We then assessed T cell proliferation by measuring the expression of Ki-67 on CD4^+^ and CD8^+^ T cells. While no significant difference could be established with regard to Ki-67 expression between the two groups, a trend toward a lower increase in the levels of Ki-67-expressing T cells was observed in animals receiving RFX+SFZ during the acute infection (Figs 3c, 3d and S3).

Altogether, these results indicate that therapy with RFX+SFZ moderately and transiently reduced the levels of systemic immune activation in acutely SIV-infected PTMs.

### Antibiotic and antiinflammatory therapy initiated during acute SIV infection significantly reduced levels of systemic inflammation in SIVsab-infected PTMs

Microbial components are known as potent triggers of inflammatory responses by numerous lymphoid and nonlymphoid cells. We therefore assessed the effect of RFX+SFZ therapy on systemic proinflammatory responses in SIVsab-infected PTMs by comparing the levels of a variety of cytokines/chemokines in plasma collected from treated and untreated PTMs.

**Fig 4:**
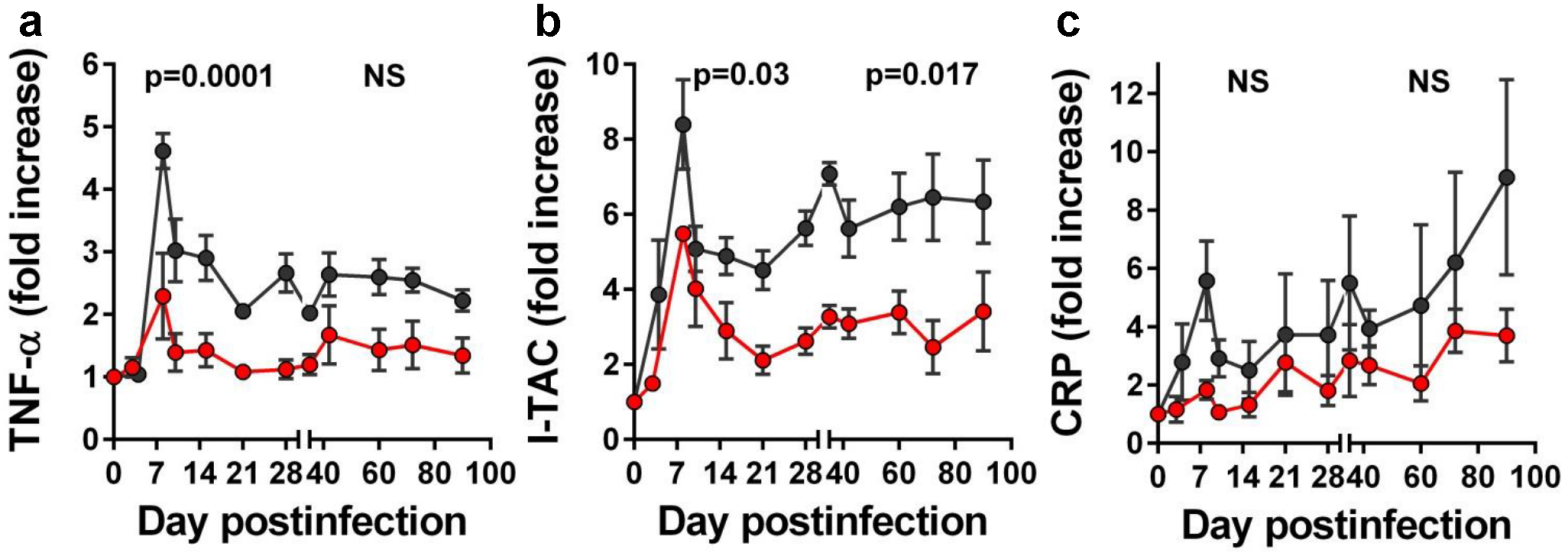
Rifaximin (RFX) + sulfasalazine (SFZ) therapy impacts the levels of inflammation during acute and early chronic SIVsab infection of pigtailed macaques (PTMs). Levels of proinflammatory cytokines were consistently lower in SIVsab-infected PTMs receiving RFX+SFZ (red) compared to untreated controls (black): (a) TNF-α; (b) I-TAC; and (c) C-reactive protein (CRP). Shown are the average values for each group and standard error of means.

RFX/SFZ therapy resulted in a reduction of proinflammatory cytokine and chemokine responses (Figs 4 and S4), as illustrated here for TNF-α (Fig 4a), and I-TAC (Fig 4b).

We also monitored C-reactive protein (CRP), a marker of acute inflammation found to predict mortality in HIV-infected patients by the INSIGHT/SMART trial [36]. CRP levels were lower in PTMs receiving RFX+SFZ, but this did not reach significance due to high variability among animals (Figs 4c and S4).

Our results indicate that RFX+SFZ therapy transiently reduced inflammatory responses in SIVsab-infected PTMs.

### Impact of the RFX+SFZ treatment on early SIVsab infection in PTMs

We next assessed the clinical impact of our therapeutic approach on early stages of SIVsab infection.

**Fig 5:**
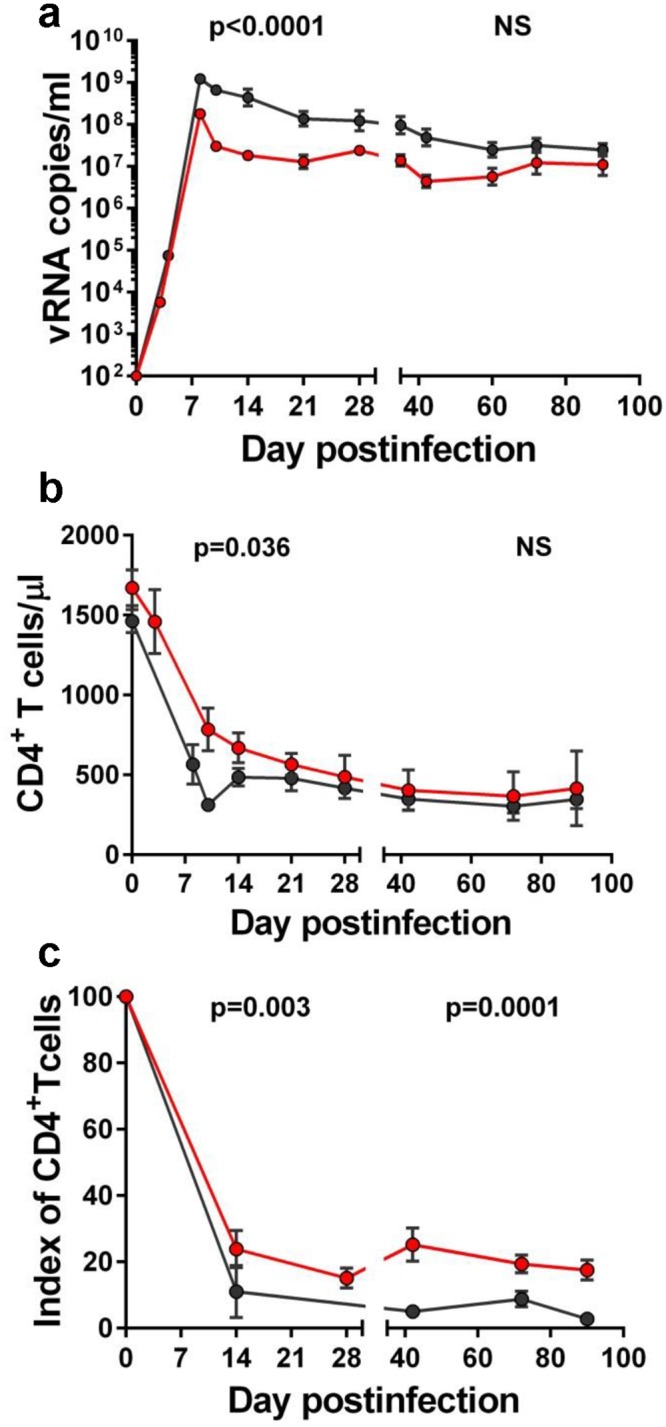
Rifaximin (RFX) + sulfasalazine (SFZ) therapy improves the natural history of SIVsab infection during acute and early chronic SIVsab infection of pigtailed macaques (PTMs). Levels of acute viral replication were significantly lower in SIVsab-infected PTMs receiving RFX+SFZ (red) compared to untreated controls (black) (a). While no significant impact of RFX+SFZ treatment could be observed in circulating CD4^+^ T cells (b), a less prominent depletion of mucosal CD4^+^ T cells could be observed in treated PTMs (c). The average values for each group and standard error of means are shown.

The levels of viral replication were significantly impacted by administration of RFX+SFZ during acute infection, when the treated animals received a higher dose of SFZ, and acute VLs in the treated group were significantly lower than those in the control group (p<0.0001) (Figs 5a and S5a). During the chronic infection, this effect waned, as the levels of viral replication were not statistically different between the two groups (Figs 5a and S5a).

Since a causal relationship between systemic immune activation and CD4^+^ T cell loss was previously reported [37–40], we compared absolute counts and percentages of CD4^+^ T cells in blood and intestine between treated and untreated PTMs at key times postinfection.

We found somewhat lower levels of peripheral CD4^+^ T cells in untreated *vs*. treated PTMs (p = 0.036) (Figs 5b and S5b). Importantly, this was also the case if we normalized each animal’s peripheral CD4^+^ T cells by its initial values (p = 0.011 for the fold change). This difference in the CD4^+^ T cell counts was confirmed by analyses of mucosal sites, where there was a tendency for better preservation of CD4^+^ T cells in PTMs receiving RFX+SLZ therapy compared to controls (Figs 5c and S5c) (p = 0.003 and p = 0.0001, for acute and chronic infection, respectively). Our results suggest that even a partial reduction of inflammation helps to better preserve mucosal CD4^+^ T cells. Note however, that the nature of this pilot project in which the follow-up was relatively short prevented us from assessing the long-term impact of RFX+SFZ therapy on mucosal CD4^+^ T cell restoration, which occurs later during SIVsab infection in PTMs [17].

### Control of microbial translocation-induced immune activation improves coagulation status in SIVsab-infected PTMs

**Fig 6:**
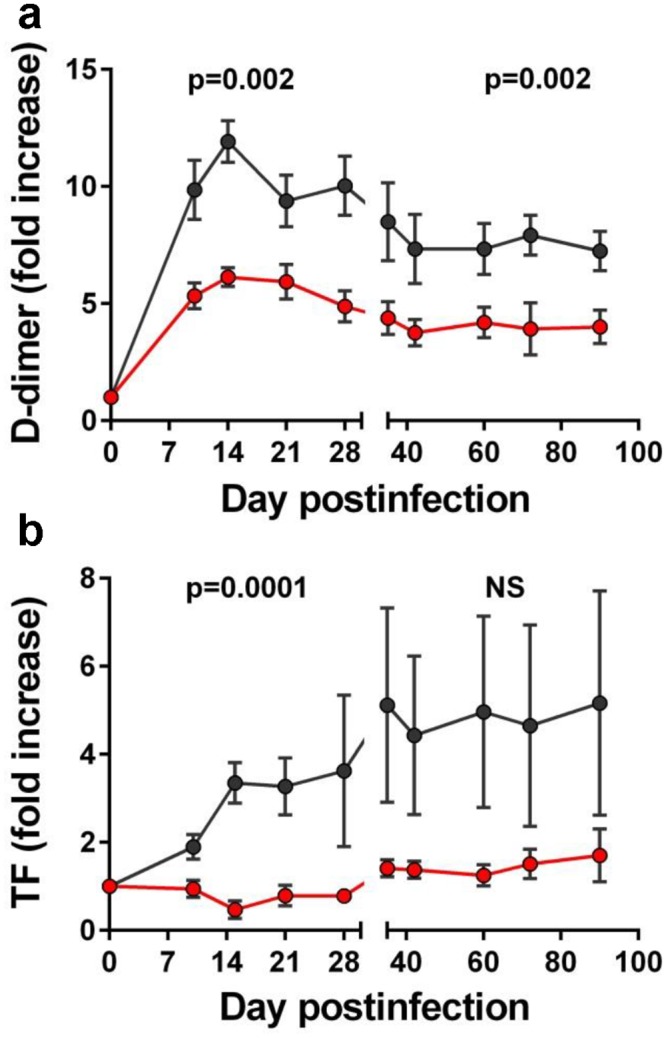
Rifaximin (RFX) + sulfasalazine (SFZ) therapy reduces the levels of coagulation biomarkers during acute and early chronic SIVsab infection of pigtailed macaques (PTMs). Levels of D-dimer (a) and tissue factor (b) were significantly lower in SIVsab-infected PTMs receiving RFX+SFZ (red) compared to untreated controls (black). Shown are the average values for each group and standard error of means.

Recent studies have suggested a strong association between hypercoagulability and inflammation during both HIV and SIV infections [29,36,41]. Therefore, we compared the levels of coagulation marker D-dimer (2-DD) in treated *vs*. untreated PTMs and report that administration of RFX+SFZ resulted in a significant reduction of 2-DD levels throughout the treatment (Figs 6a and S6) (p = 0.002 for both acute and chronic infection).

It has been proposed that the mechanism responsible for the hypercoagulable status observed in HIV patients involves tissue factor (TF) overexpression in response to microbial products [42,43]. As such, we measured plasma soluble TF (sTF) levels in RFX+SLZ-treated PTMs and compared them to those observed in untreated PTMs. We report that, while a 3–4 fold increase in sTF expression occurred during acute SIVsab infection in untreated PTMs relative to the control group, plasma levels of sTF remained virtually unchanged from the baseline in PTMs that received RFX+SFZ therapy (Figs 6b and S6) (p = 0.0001 for the change in TF levels in acute infection, and p = 0.055, for the difference of those levels in chronic infection).

Our results thus reinforce the role of microbial translocation-induced immune activation in development of cardiovascular comorbidities associated with HIV/SIV infection.

### RFX+SFZ administration to chronically SIVsab-infected, ARV-naïve PTMs did not significantly impact microbial translocation, natural history of SIVsab infection and comorbidities

Our results showed that therapy with RFX and SFZ administered early during SIVsab infection of PTMs had a higher efficacy during the acute stage of infection (Figs 1–6).

**Fig 7:**
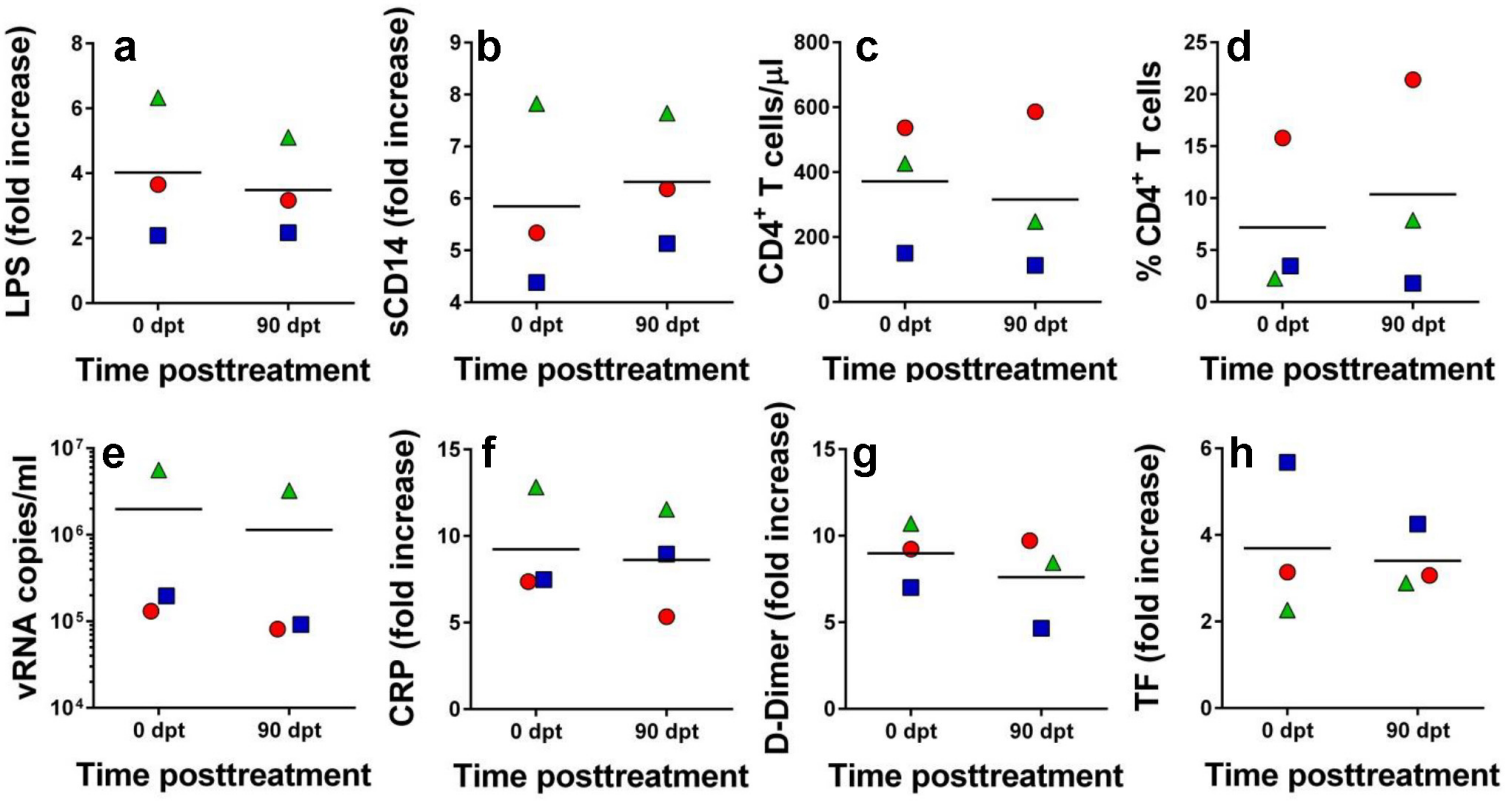
Rifaximin (RFX) + sulfasalazine (SFZ) therapy have no impact on the natural history of SIVsab infection in chronically SIVsab-infected pigtailed macaques. Combination RFX+SFZ therapy did not impact microbial translocation, as illustrated by the plasma levels of LPS (a) or sCD14 (b), peripheral CD4^+^ T cell counts (c), mucosal CD4^+^ T cell levels (d), viral replication (e), inflammation (C-reactive protein) (f) or coagulation, as illustrated by the levels of D-dimer (g) and tissue factor (h). The fold increases from the preinfection levels of the tested biomarkers are shown. Treatment was initiated at 170 days after SIV infection, when biomarkers were already increased from the preinfection baseline. A therapeutic effect at the end of treatment (90 dpt) would have been illustrated by normalization of the increases from the baseline observed at treatment initiation (0 dpt).

The vast majority of HIV infected patients are, however, in the chronic stage, when the mucosal lesions are extensive and thus microbial translocation from the lumen fuels high levels of immune activation. It is therefore likely that in chronically-infected patients the vicious circle of HIV pathogenesis is more difficult to break, as suggested by previous experience with ART, which cannot completely control immune activation, in spite of providing long-term control of virus replication [19]. We therefore assessed the efficacy of the RFX+SFZ therapy in a small group of chronically SIVsab-infected PTMs. Three animals were included in this study. They received the same combination of RFX+SFZ for 90 days (starting from 170 dpi). Despite of using the high SFZ dose, this treatment had no discernible effect on key parameters of SIV infection. Thus, RFX+SFZ therapy did not have any impact on microbial translocation, as monitored by plasma levels of LPS (Fig 7a) and sCD14 (Fig 7b). No effect could be observed on peripheral (Fig 7c) or mucosal (Fig 7d) CD4^+^ T cells, viral loads (Fig 7e), or on the levels of systemic inflammation as illustrated by the virtually unchanged levels of CRP (Fig 7f). Finally, administration of RFX+SFZ therapy to chronically-infected PTMs had no impact on the coagulation markers, neither on 2-DD (Fig 7g) nor on TF (Fig 7h). Our results thus show that breaking the negative chain of events characteristic to SIV/HIV pathogenesis is more difficult during chronic infection than during the early stages that precede the onset of mucosal injuries. Note however, that, unlike HIV-infected patients, the majority of which are on ART, our RFX+SFZ study used ART-naïve NHPs.

### RFX+SFZ administration to uninfected, ARV-naïve PTMs altered the fecal microbiome

**Fig 8:**
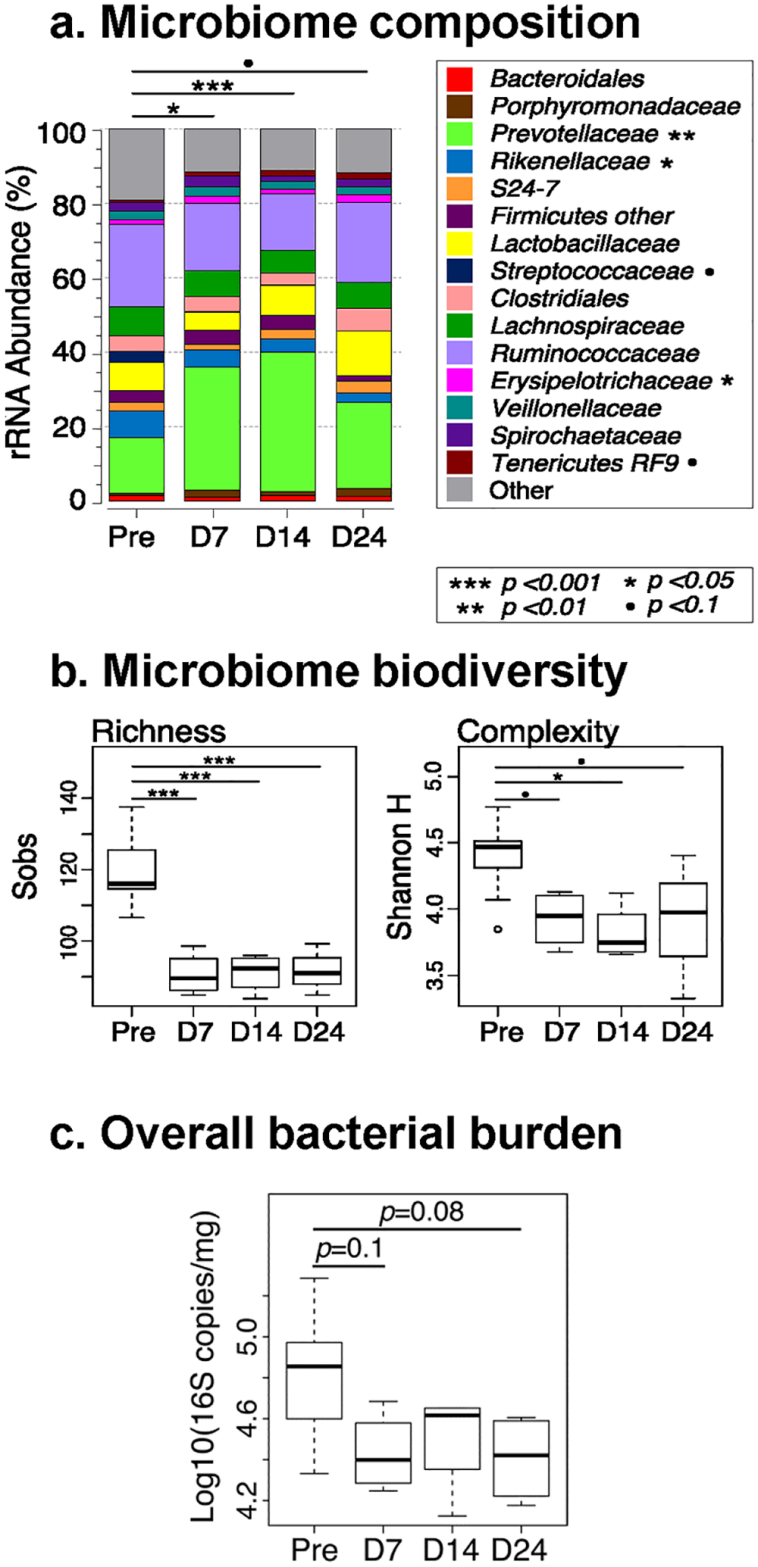
Rifaximin (RFX) treatment alters the gut microbiome. Broad-range 16S rRNA amplification and high-throughput sequencing was used to profile fecal microbiota before (Pre), during (D7, D14), and following (D24) treatment, as described in the text. (a) Summary of the family-level distribution of bacterial taxa (expressed as percent relative abundance) averaged across each group. Lines and symbols above the stacked bars indicate significant differences in microbial composition assessed by PERMANOVA tests of Bray-Curtis similarity scores. Individual taxa that differed significantly in abundance across timepoints, as assessed by Kruskal-Wallis tests, are indicated in the legend. (b) Comparison of two measures of biodiversity (richness and complexity), inferred from 16S rRNA sequence datasets, between timepoints. Significance was assessed by Welch Two Sample t-test. (c) Boxplots summarizing the log10-transformed bacterial loads measured in fecal samples by 16S rRNA gene PCR and normalized to fecal weight. Bacterial loads differed across all four timepoints (ANOVA p = 0.04). Pairwise comparisons with P-values less than 0.1 are indicated.

As in our original study we did not collect fecal samples to assess the impact of RFX treatment on the gut microbiome, we treated 4 SIV-naive PTMs with the same dose of RFX and comprehensively profiled fecal bacterial communities in serial fecal samples using high-throughput 16S rRNA sequencing. RFX was administered for two weeks. The fecal samples were collected before treatment initiation, at 7 and 14 dpt, as well as at 14 days after RFX treatment cessation. Our results demonstrated that RFX administration significantly shifted the composition of the fecal microbiota at treatment days 7 (p = 0.006 PERMANOVA test) and 14 (p<0.001 PERMANOVA test), relative to baseline (Fig 8a). These differences were most evident in >2 fold expansion in relative abundance of *Prevotellaceae* in the treatment groups at 7 dpt (p = 0.003; median values of 14.9% and 31.0% at the baseline and 7 dpt, respectively) and 14 dpt (p = 0.003; median values of 14.9% and 37.4% at the baseline and 14 dpt, respectively). Treatment also significantly reduced fecal biodiversity, as demonstrated by the significantly altered richness and complexity indices at 7 and 14 dpt, compared with baseline (Fig 8b).

Although samples collected at 14 days posttreatment interruption also differed from baseline in microbiota composition (p<0.053; Fig 8a) the differences appeared to be less drastic than those observed between baseline and 7 and 14 dpt samples. For example, the median *Prevotellaceae* abundance at 24 dpt was no longer significantly different from baseline (p = 0.15; median values of 14.9% *vs*. 25.6% at the baseline and 24 dpt, respectively). Nevertheless, both richness and complexity indices remained significantly reduced, compared to the baseline.

To determine whether RFX treatment altered the fecal bacterial loads, we assayed all specimens using a pan-bacterial 16S rRNA QPCR assay and normalized results to the wet weights of the fecal aliquots that were extracted. Although average bacterial loads were reduced to 38–47% of the baseline values in all three postbaseline timepoints (overall p = 0.04), only the differences between baseline and 7 dpt (p = 0.08) and 24 dpt (p = 0.1) approached significance (Fig 8c).

## Discussion

In this study we showed that administration of an intraluminal antibiotic (RFX) and a gut-focused anti-inflammatory drug (SFZ) transiently improved the natural history of the early stages of SIVsab infection in PTMs. In NHPs receiving this treatment, a partial control of microbial translocation, immune activation and inflammation could be documented, resulting in a transient amelioration of their deleterious consequences, such as CD4^+^ T cell depletion or the procoagulant state. Therefore, our results support therapies aimed at controlling immune activation as effective approaches to improve the outcome of HIV/SIV infection.

The rationale for such a therapeutic approach for HIV/SIV infections is based on the current paradigm of AIDS pathogenesis [1]. In this paradigm, upon infection, activation of target cells and massive acute viral replication induce inflammation in the gut. At this site, massive immune and epithelial cell death occur due to virus depletion of target cells and activation-induced bystander apoptosis. Cell death and inflammation alter the mucosal barrier and result in translocation of microbial elements from the intestinal lumen to the general circulation [1,22,23,25,34]. The main consequence of microbial translocation is systemic immune activation, which further increases target cells for the virus, thus fueling virus replication and additional immune activation. This vicious circle in which each process fuels the others ultimately results in a complete exhaustion of the immune system and death. In support of this paradigm are not only studies demonstrating that high viral replication and mucosal CD4^+^ T cell depletion that triggers this deleterious chain of events occur very early in the infection [44–47], but also the results of clinical studies (i.e., SMART/INSIGHT) showing that biomarkers of inflammation and microbial translocation are associated with increased risk of death [36,41]. While most of the results in the field converge and support this paradigm, the vast majority of available data is correlative.

Some authors contest a central role of microbial translocation in the pathogenesis of AIDS, arguing that it is not possible to clearly establish whether or not microbial translocation is a cause or a consequence of generalized immune activation [48–52]. Here, rather than directly testing the primordiality of microbial translocation over inflammation (or *vice versa*) in driving disease progression, we designed a therapeutic approach consisting of drugs targeting both mucosal inflammation (SFZ) and intestinal microbial burden (RFX). We reasoned that this approach would result in a reduction of microbial translocation by either limiting mucosal breaches or by limiting availability of microbial products, thus reducing immune activation and comorbidities. Rather than targeting a specific mechanism of generalized immune activation, our intervention focused on breaking the vicious circle of SIV pathogenesis that leads to persistent systemic immune activation, comorbidities, and ultimately, disease progression.

We employed our SIVsab-infected PTM model of HIV infection [17]. The reason for choosing this model over the classical SIVmac239-infected rhesus macaque model is that PTMs are more prone to mucosal dysfunction and consequently may be a better system to model interventions aimed at controlling mucosal inflammation and microbial translocation [16,17,29,53,54]. Moreover, PTMs better reproduce HIV-associated comorbidities than rhesus macaques [29,55].

To reduce bacterial load in the intestinal lumen and thus diminish microbial translocation into general circulation we employed RFX, a semisynthetic derivate of rifamycin with broad activity against Gram^neg^, Gram^pos^, *Mycobacteria* and anaerobes which significantly decreases gastrointestinal flora [56]. The rationale for choosing RFX to control intestinal flora is that it has a very limited absorption into blood stream after oral administration [31,56]. The lack of RFX absorption explains both drug efficacy and safety in treating enteric infections. Plasma levels of RFX being negligible, bacteria are not exposed to selective pressures outside the gastrointestinal tract, and thus bacterial resistance to RFX (one of the major concerns with antibiotics administration) is low [31]. RFX is effective in patients with microbial translocation-associated hepatic encephalopathy [31,56] and is safe for long-term administration. To reduce intestinal inflammation we administered SFZ, an antiinflammatory sulfa drug derivate of mesalazine. SFZ decreases the synthesis and/or release of leukotrienes, platelet activating factor, IL-1 and IL-2, and inhibits TNF-α signaling and HLA-DR expression in colonic epithelial cells and the levels of reactive oxygen species in neutrophils [57]. The drug was previously used in patients with inflammatory bowel disease, including ulcerative colitis and Crohn’s disease, both of which are associated with elevated levels of microbial translocation [32,33,58].

To assess the effects of RFZ+SFZ therapy on SIV pathogenesis without additional confounding factors, we did not administer ART to SIVsab-infected PTMs receiving RFX+SFZ. Meanwhile, to maximize the clinical effects, we initiated RFX+SFZ therapy at the time of PTM challenge with SIVsab. Two rationales stand behind this approach. First, this design allowed administration of the RFX+SFZ therapy prior to mucosal damage inflicted by high levels of viral replication and initial depletion of mucosal CD4^+^ T cells. Second, during the acute and early chronic stages of SIV infection, there are multiple and dramatic changes in biomarkers associated with immune activation, and thus therapeutic impact can more readily be detected. In a second step, we administered the same RFX+SFZ therapy to a subset of chronically SIVsab-infected, ART naïve PTMs to compare therapeutic efficacy before and after occurrence of the mucosal damage inflicted by SIVsab. One may argue that administration of the RFX+SFZ therapy should have been performed in the chronic stage of infection, under ART and that given the fact that we administered a combination of drugs; we cannot clearly conclude which of the two drugs had a more prominent effect on microbial translocation. However, we reasoned that should we document a benefit of this therapeutic approach in this system, we could then embark on additional animal studies designed to more realistically reproduce the vast majority of HIV-infected patients, who receive ART and are in more advanced stages of infection.

Administration of the RFX+SFZ therapy to acutely SIV-infected PTMs clearly impacted microbial translocation, as shown by plasma levels of both LPS and sCD14 in treated PTMs compared to untreated controls. IHC assessment of LPS levels in peripheral LNs confirmed that the number of LPS positive macrophages was significantly lower in treated PTMs during the first 42 dpi.

A key result of the study was that, paralleling the lower levels of microbial translocation, the levels of immune activation and inflammation biomarkers were also significantly reduced in treated PTMs.

Probably as a result of a better control of immune activation, a trend toward better preservation of mucosal CD4^+^ T cells was observed in PTMs treated with RFX+SFZ. One may argue that the overall impact of the RFX+SFZ therapy on CD4^+^ T cells was very limited. This result might be due to the fact that our therapeutic approach only marginally reduced acute viral replication, which is responsible for the massive acute depletion of mucosal memory CD4^+^ T cells characteristic of HIV/SIV infection [26,27,44–47]. Also, the follow-up was relatively short, preventing us from assessing a putative positive impact on CD4^+^ T cell restoration. Nevertheless, our results point to a clear therapeutic benefit of controlling microbial translocation in SIV-infected NHPs.

Finally, RFX+SFZ therapy resulted in a significant reduction in levels of sTF and of coagulation biomarker 2-DD in PTMs receiving RFX+SFZ compared to controls. Strong correlations between microbial translocation and coagulation markers were previously reported to occur in both progressive and nonprogressive HIV/SIV infections [29,36]. This is not surprising, as microbial products can trigger increased TF expression on immune cells [42]. TF (thromboplastin) being a major activator of the coagulation cascade, its increase during progressive HIV/SIV infections may explain the generation of the prothrombotic status described in chronic HIV/SIV infection. In a clinical trial targeting microbial translocation with sevelamer, an effect on sTF was reported [43]. Our study provides further evidence that interventions targeting microbial translocation reduce TF and positively impact coagulation.

It was previously reported that high persistent levels of systemic inflammation are related to the development of comorbidities and premature aging in HIV-infected patients and SIV-infected NHPs [29,36,59]. Also, previous studies showed that 2-DD is strongly and independently linked to cardiac events and death [29,36,59]. We also report that RFX+SFZ therapy impacted the levels of 2-DD and TF, and we concluded that therapies aimed at reducing microbial translocation may have a potential impact on HIV/SIV-associated noninfectious comorbidities.

Since we have employed more than a single drug, we cannot conclude at this stage whether the observed effects are being mediated by the presumed reduction in enteric bacterial burden mediated by RFX or by the anti-inflammatory effects of SFZ, or by both. Brenchley et al., [24] demonstrated that treatment of SIV infected macaques with antibiotics could reduce plasma LPS. Furthermore, prolonged antibiotic treatment with co-trimixazole successfully reduced the markers of microbial translocation [60]. It thus seems reasonable that the reduction in plasma LPS and systemic activation could be a result of antibiotic treatment. On the other hand, eicosanoids, which are modulated by sulfasalazine, have been reported to have direct anti HIV effects [61,62], as well as potential secondary effects through effects on immune cell activation [63]. Administration of mesalamine, a related anti-inflammatory drug to immunocompetent patients reduced diarrhea and mucosal inflammation [64]. Thus, both drugs could have contributed to the observed effects.

A disappointing outcome of our study is that the therapeutic effect was only transient, which will likely limit its applicability as a therapy in HIV-infected patients. While we do not have a definitive explanation for the waning of therapeutic efficacy, there are several potential causes for the observed results. First, a limited efficacy of RFX, due to: (i) its inability to decrease the overall bacterial load; (ii) emergence of dysbiotic bacteria under RFX treatment; and/or (iii) emergence of microbial resistance to RFX. Due to the fact that we did not analyze the microbiome of the RFX-treated PTMs, we cannot definitively opt for any of these scenarios. It was reported that RFX does not induce significant changes in microbial abundance [65]. Based on the reported data and to determine whether or not RFX has any impact on the intestinal microbiome, we designed a study to comprehensively profile fecal bacterial communities in serial samples from 2-week RFX-treated, uninfected PTMs. We report that administration of RFX significantly altered richness and complexity of the intestinal flora in uninfected NHPs, thus directly documenting a clear therapeutic effect of RFX. Fecal bacterial loads were reduced ~2 fold even after Rifaximin treatment ended. Although treatment did not dramatically alter bacterial load, the composition and diversity of the fecal microbiota differed significantly during and after Rifaximin treatment. Our experiment was, however, short and cannot confirm that the therapeutic effect can be maintained at long term. During previous administration of antibiotic therapy to control microbial translocation in chronically SIV-infected RMs, a loss of the therapeutic effect was reported after few weeks of therapy [24], so we cannot exclude this scenario for our current study.

The offset of the antibiotic efficacy may be a consequence of the fact that the antibiotic therapy can only partially reduce the microbial flora of the gut, but cannot prevent the breaches in the intestinal wall from occurring during HIV infection. Once the lesions produced, disrupting the vicious circle of HIV/SIV pathogenesis becomes a much more difficult task to achieve and, as such, based on both our results as well as reported data from human and macaque trials [24,66], it is to be expected that antibiotic therapy, especially a short term one, will have only a transient and limited impact on the levels of microbial translocation observed in HIV infection. Corroborated with the need to keep microbial translocation at bay indefinitely and with the long term drug toxicity, this observation points to the relatively limited applicability of this therapeutic strategy in HIV-infected patients. A second reason for the loss of therapeutic effect might have been the reduction in the SFZ dose that was operated after 30 days of treatment, at the end of the acute infection. The rationale behind the dose reduction was that the high levels of acute inflammation are partially resolved at the end of acute infection, which might have allowed us to pass to a maintenance SFZ dose, similar to the strategy used in patients with inflammatory bowel disease. Finally, therapeutic efficacy may have been impacted by a more extensive gut damage occurring at later stages of infection. This might have limited the ability of the two drugs to control the mucosal lesions and the levels of translocated microbial products.

To assess whether the therapeutic effect is different during different stages of SIV infection, we administered RFX+SFZ (high dose) to three late chronically-infected PTMs, and report that this approach did not have a discernible effect on the natural history of chronic SIV infection. While these results have to be interpreted with extreme caution due to the small sample size of the study group, the differences in treatment efficacy between acutely-infected and chronically-infected PTMs suggest that the degree of gut damage at the initiation of treatment is a key determinant of the therapeutic success. Furthermore, since in this experiment we employed the high dose of SFZ, we can conclude that the dose of SFZ is likely not responsible for the loss of therapeutic efficacy observed at the passage from acute to chronic infection. These results corroborate the results of previous studies that reported a modest therapeutic response after a short course treatment with RFX in HIV-1 infected patients on ART [66]. The results of the two studies are not, however comparable, because the human trial did not utilize an anti-inflammatory drug, while our NHP study did not achieve virus control with ART. The only common point of the two studies was that they both employed severely immune suppressed subjects (immune nonresponder HIV patients *vs*. late chronically-infected ART naïve PTMs).

While a clear effect was documented after administration of RFX+SFZ, it is unlikely that such a therapeutic approach can be employed in HIV-infected patients. First, treatment efficacy was transient even in this study design in which RFX+SFZ were initiated very early postinfection, prior to the occurrence of the gut damage. Second, clinical trials, as well as our results here, showed that RFX administration during chronic HIV/SIV infection has only marginal effects [66]. Third, long-term antibiotic treatment may induce dysbiosis that can be detrimental for HIV-infected patients, for which improvement of the gut flora with probiotics is beneficial [67]. Finally, prolonged treatment with anti-inflammatory drugs may boost cardiovascular disease that is already problematic in HIV patients [36,68].

As such, one may downplay the therapeutic endpoint of this study and of this strategy to control microbial translocation in general. However, our results are relevant for understanding the pathogenesis of HIV/SIV infection as we demonstrate that limiting the intestinal dysfunction and control of microbial translocation even for a short period of time contribute to a significant reduction of the immune activation. Thus, our study confirm our previous results with sevelamer administration to SIVsab-infected PTMs [16]. It also complements other approaches in the field aimed at directly modulating inflammatory responses during acute SIV infection [69]. While some of these approaches may fail to improve the outcome of infection, they all point to a critical need to better understand the nature and the sources of inflammation during HIV/SIV infection in order to guide anti-inflammatory therapies which otherwise may be proven harmful.

To achieve these goals, the use of NHP models is critical and warranted by the possibility to perform interventions at well-defined time points of infection, in a refined system and without any interference of multiple behavioral risk factors for comorbidities.

## Materials and Methods

### Ethics statement

Fifteen male PTMs were included in this study. All animals were housed and maintained at the RIDC Park animal facility of the University of Pittsburgh according to the standards of the Association for Assessment and Accreditation of Laboratory Animal Care (AAALAC), and experiments were approved by the University of Pittsburgh Institutional Animal Care and Use Committee (IACUC) (IACUC protocol: #09039, approved in 2009). The animals were fed and housed according to regulations set forth by the *Guide for the Care and Use of Laboratory Animals* and the Animal Welfare Act [70]. All PTMs included in this study were socially housed (paired) indoors in stainless steel cages, had 12/12 light cycle, were fed twice daily, and water was provided ad libitum. A variety of environmental enrichment strategies were employed including housing of animals in pairs, providing toys to manipulate and playing entertainment videos in the animal rooms. In addition, the animals were observed twice daily and any signs of disease or discomfort were reported to the veterinary staff for evaluation. For sample collection, animals were anesthetized with 10 mg/kg ketamine HCl (Park-Davis, Morris Plains, NJ, USA) or 0.7mg/kg tiletamine HCl and zolazepan (Telazol, Fort Dodge Animal Health, Fort Dodge, IA) injected intramuscularly. The animals were sacrificed by intravenous administration of barbiturates prior to the onset of any clinical signs of disease.

### SIV challenge and treatment

All animals were intravenously infected with plasma equivalent to 300 tissue culture infectious doses (TCID50) of SIVsabBH66 (and containing 10^7^ vRNA copies/ml). At the time of virus inoculation, five PTMs received therapy with Rifaximin (400 mg/day orally) and Sulfasalazine (dosed at 75 mg/kg orally for the first month of treatment, then adjusted to 25 mg/kg for the following 2 months). Total duration of therapy was 3 months. The remaining PTMs (n = 7) were grouped as untreated SIVsab-infected controls in which infection followed its natural course.

### Blood and tissue sampling

Blood was collected from all PTMs prior to infection (day -15 and day 0), during acute SIVsab infection (3, 8, 10, 14, 21, and 28 dpi), around the viral set-point (35 and 42 dpi), and during chronic infection (60, 72, and 90 dpi) (S1 Fig).

Intestinal biopsies were collected prior to infection, during acute infection (14 and 28 dpi) and during chronic infection (42, 72, and 90 dpi), as previously described [30,71–73] (S1 Fig).

Within one hour after blood collection, plasma was harvested and peripheral blood mononuclear cells (PBMCs) were separated from the blood using Ficoll density gradient centrifugation. Lymphocytes from the intestine were isolated and stained for flow cytometry, as previously described [30,71–73]. Intestinal biopsies were processed as described previously to obtain an enriched mononuclear cell suspension. Briefly, intestinal samples were minced mechanically, washed with EDTA and subjected to collagenase digestion, followed by Percoll density gradient centrifugation [30,71–73].

### Plasma viral load quantification

SIVsabBH66 viral RNA (vRNA) loads were quantified by real-time PCR, as described previously [72–74].

### Antibodies and flow cytometry

Whole blood and mononuclear cells isolated from intestinal biopsies were stained for flow cytometry using a six-color technique as described previously to assess changes in the levels of major T cell populations and their immune activation status. The mAb combination used was: CD3-Pacific Blue, CD4-allophycocyanin, CD8-Texas Red, HLA-DR-allophycocyanin-Cy7, CD38-PE (BD Biosciences) and Ki-67–FITC (BD Pharmingen). All Abs were validated and titrated using PBMCs from PTMs [17,75]. Samples were stained for Ki-67 using the Ki-67/FITC–conjugated mouse anti–human mAb set (BD Pharmingen) as per the manufacturer’s instructions. Stained cells were analyzed with an LSRII flow cytometer (BD Biosciences) and FlowJo Version 7.6 software (TreeStar). CD4^+^ and CD8^+^ T cell percentages were obtained by first gating on lymphocytes, then on CD3^+^ T cells. Activation markers were determined by gating on lymphocytes, then on CD3^+^ T cells, and finally on CD4^+^CD3^+^ or CD8^+^CD3^+^ T cells.

### Assessment of the levels of microbial translocation

Plasma levels of LPS were measured as previously described [24]. Several factors present in plasma have been shown to interfere with LPS measurements (LBP, EndoCAb, HDL, plasma turbidity, proteins and triglycerides). Therefore, to minimize any possible interference, plasma samples were diluted 5 fold with endotoxin-free water and then heated to 85°C for 15 min to inactivate plasma proteins. Plasma LPS was quantified with a commercially available *Limulus* amebocyte lysate assay (Cambrex), according to manufacturer’s protocol. Each sample was run in duplicate.

Plasma-soluble CD14 (sCD14) levels were measured as a surrogate marker of microbial translocation [24]. CD14 is a transmembrane protein which also exists in soluble form (sCD14; both as a shed membrane form and an alternatively spliced form), as a part of the complex that presents endotoxin (lipopolysaccharide-LPS) to TLR4 on monocytes. When monocytes are activated, ectodomain shedding results in increased sCD14 levels. sCD14 is therefore surrogate for direct measurement of endotoxin or Gram negative bacteria which translocate from the intestinal lumen to the general circulation [24,29,30]. sCD14 levels were measured using a quantitative sandwich enzyme immunoassay technique (Quantikine Human sCD14 Immunoassay, R&D Systems, Minneapolis, MN). The detection limit of this kit is 200 ng/mL and can range up to 5000 ng/mL at a dilution factor of 1:200, with an interassay coefficient of variability of 7.19% to 10.9%.

### Immunohistochemistry for LPS

Immunohistochemical (IHC) analysis of LPS was performed on formalin-fixed, paraffin-embedded tissue samples, as described [16]. Four μm-thick sections were deparaffinized, rehydrated, and rinsed. For antigen retrieval, the sections were microwaved in Vector Unmasking Solution (Vector Laboratories Burlingame, CA, USA) and treated with 3% hydrogen peroxide. Sections were incubated with LPS (Hycult Biotech, USA) monoclonal primary antibody at a 1:100 dilution. Secondary antibodies and Avidin/Biotin complex were from the Vector Vectastain ABC Elite Kit. For visualization, sections were treated with DAB (Dako Carpinteria, CA. USA), counterstained with hematoxylin, dehydrated, and mounted in a xylene-based mounting media.

Quantification was performed using open source FIJI image software using 10 images, per section, per time point, per animal. The positive signal was isolated via color threshold; percent area positive was measured and averaged.

### Testing of soluble markers of inflammation

Cytokine testing in plasma was done using a sandwich immunoassay-based protein array system, the Cytokine Monkey Magnetic 28-Plex Panel (Invitrogen, Camarillo, CA), as instructed by the manufacturer. Results were read by the Bio-Plex array reader (Bio-Rad Laboratories, Hercules, CA), which uses Luminex fluorescent-bead-based technology (Luminex Corporation, Austin, TX). The analysis was focused on proinflammatory cytokines.

### C reactive protein (CRP) testing

CRP is an acute-phase protein which rises in the plasma in response to inflammation. It was first identified in the serum of patients with acute inflammation that reacted with the C-polysaccharide of *Pneumococcus*. CRP binds to phosphocholine expressed on the surface of dead cells and some types of bacteria, in order to activate the complement system via the C1Q complex. The SMART trial identified CRP as one of the biomarkers associated with death in HIV-infected patients [36]. CRP was measured using a monkey CRP ELISA kit (Life Diagnostics, PA) as per manufacturer recommendations.

### Coagulation marker testing

Coagulation status was estimated by measuring plasma levels of 2-DD. 2-DD is a terminal product of plasmin acting on a fibrin clot that increases during coagulation, disseminated intravascular coagulation, and deep vein thrombosis. 2-DD was reported to independently correlate with lentiviral disease progression and death in HIV-infected patients [36] and SIV-infected macaques [29]. 2-DD was measured using a STAR automated coagulation analyzer (Diagnostica Stago) and an immunoturbidimetric assay (Liatest D-DI;Diagnostica Stago). The analytical coefficient of variation ranged from 5%-14%.

### Tissue factor (TF) testing

TF is a transmembrane cell-surface glycoprotein known for its role in initiating coagulation. Once TF complexes with factor VII, it can initiate both intrinsic and extrinsic pathways of coagulation. TF increase in plasma indicates a procoagulant environment and is found in patients diagnosed with malignant solid tumors [76,77]. In HIV-infected patients, monocyte expression of TF is correlated with HIV levels in plasma, immune activation, and plasma levels of sCD14 [42]. TF levels also correlate with plasma levels of 2-DD, reflective of *in vivo* clot formation and fibrinolysis [42]. TF levels in plasma were tested by IMUBIND Tissue Factor ELISA (Sekisui Diagnostic, Lexington, MA) based on the manufacturer’s instructions.

### Microbiome analysis

DNA extractions from ~100 mg of stools were performed using the UltraClean Fecal DNA kit (MoBio Laboratories, Inc., Carlsbad, CA) per the manufacturer’s protocols. Preparation, high-throughput sequencing, and taxonomic classification of broad-range bacterial 16S rRNA amplicon libraries followed our previous work [78–80]. In brief, PCR amplicons were generated using primers that target approximately 300 base pairs of the V1V2 variable region of the 16S rRNA gene using primers 27F-YM (5’ AGAGTTTGATYMTGGCTCAG) [81] and 338R (5’ TGCTGCCTCCCGTAGGAGT) [82]. Illumina paired-end sequencing was performed on the Miseq platform with version v2.3.0.8 of the Miseq Control Software and version v2.3.32 of MiSeq Reporter, using a 600-cycle version 3 reagent kit. Paired-end reads were assembled then aligned and classified with SINA (1.3.0-r23838) [83] using the 479,726 sequences in Silva 115NR99 [84] as reference configured to yield the Silva taxonomy. A total of 4,475,358 high-quality 16S sequences were generated, with a median of 192,833 sequences/sample (range: 141,630–365,560). Operational taxonomic units (OTUs) were produced by clustering sequences with identical taxonomic assignments. Relative abundances of OTUs were calculated for each subject by dividing the sequence counts observed for each OTU by the total number of high-quality bacterial 16S rRNA sequences generated for the subject. All sequence libraries had Goods coverage scores ≥ 99.9% at the rarefaction point of 141,630 sequences, indicating that sequence coverage was excellent. All DNA sequence data were deposited in the NCBI short read archive under Project PRJNA296587.

The Explicit sequence analysis software package (v.2.7) [85] and R-statistical package (v. 2.15.2) were used for all microbiome statistical analyses. Ecological indices of richness (S_obs_, S_chao1_), diversity (Shannon’s diversity [Ho]), evenness ([Ho/Hmax]), and coverage (e.g., Good’s index) were computed through bootstrap resampling (1000 replicates) and rarefaction of the OTU distributions obtained from each specimen. Differences in microbiome composition (i.e., OTU distributions taken as a whole) between infant subsets were quantified by the Bray-Curtis beta-diversity index using the *adonis* function of the *vegan* R package, which performs a non-parametric multivariate analysis of variance (PERMANOVA with 50,000 replicate resamplings) [86]. Individual OTUs that differed in abundance between groups were identified by Kruskal-Wallis non-parametric analysis of variance tests. Because of the exploratory nature of the microbiome analysis and the relatively small sample size, we did not correct P-values for multiple comparisons.

### Measurement of bacterial loads

Approximately 25 mg aliquots of stool were weighed, and then DNA purified using the UltraClean Fecal DNA kit (MoBio Laboratories, Inc., Carlsbad, CA). 16S rRNA gene copy numbers were measured in duplicate for each sample using a panbacterial quantitative PCR TaqMan assay [87]. 16S copy numbers were estimated by reference to a dilution series standard curve of a plasmid carrying a 16S rRNA gene [88]. Results were averaged for technical replicates and normalized by the weight of each stool aliquot. Log10-transformed data were analyzed across all four timepoints by ANOVA and pairs of timepoints by Tukey Honest Significant Difference tests.

### Statistical analysis

We compared microbial translocation, cytokine and other immune parameters between RFX+SFZ-treated and control animals at two time periods separately: acute phase and post-acute phase. To improve the power of these analyses, we used linear mixed-effects models [89]. In this approach, we use all the measurements available together, and use macaque as the grouping (or random) factor to account for the repeated measurements made in each animal. We tested multiple models with fixed effects for time and treatment, with or without interactions. In this way, we are analyzing not only differences in the levels of the variable between treated and control monkeys, but also whether there is a difference in the variability of those levels over time (corresponding to the interaction term).

During the acute phase the behavior of the different parameters assayed over time is variable, so to allow for a general pattern of dependency of the variable on time, we considered the number of days since infection (between day 1 and day 21, as the acute phase) as a categorical factor (akin to a repeated ANOVA analysis) [89]. For this, we need to have measurements on the same days for controls and treated animals. Therefore, we did not consider time points when there was only data for one of the groups. During the post-acute phase, from day 42 p.i. onwards, the values of all parameters tended to be more constant or changed monotonically, therefore, we considered the number of days since infection as a continuous variable (akin to an ANCOVA analysis).

Assumptions on the distribution of residuals and appropriateness of the fitted values were checked by visual inspection of residual and fitted plots. The best model for the data (with or without the interaction term) was chosen comparing the log likelihood. For these analyses we used the lme function of the nlme package [89] of R (http://cran.r-project.org/).

Finally, to compare the percentage of CD4^+^ T cells in the intestine, for which there was only one measurement in the acute phase for both groups (at day 14), precluding the use of mixed-effects models, we used a Mann-Whitney test.

With the exception of viral loads and peripheral CD4^+^ T-cells, we analyzed fold-changes from baseline for all variables studied. P-values<0.05 were considered to be significant.

## Supporting Information

S1 Fig Treatment and sampling schedules for the PTMs receiving the RFX+SFZ treatment and for untreated controls. (PDF)

S2 Fig Rifaximin (RFX) and sulfasalazine (SFZ) treatment reduces microbial translocation during early SIVsab infection of pigtailed macaques (PTMs). (a) Comparison between plasma LPS levels in SIVsab-infected PTMs receiving RFX+SFZ (red) and untreated controls (black). (b) Comparison between plasma sCD14 levels in SIVsab-infected PTMs receiving RFX+SFZ (red) and untreated controls (black).
(PDF)

S3 Fig Rifaximin (RFX) and sulfasalazine (SFZ) treatment impacts T cell immune activation during acute and early chronic SIVsab infection of pigtailed macaques (PTMs). Significant differences were observed between SIVsab-infected PTMs receiving RFX+SFZ (red) and untreated controls (black) with regard to CD38 and HLA-DR expression by CD4^+^ T cells (a) and CD8^+^ T cells (b). Conversely, Ki-67 expression by CD4^+^ T cells (c) and CD8^+^ T cells (d) was not statistically different between the two groups.
(PDF)

S4 Fig Rifaximin (RFX) and sulfasalazine (SFZ) impacts the levels of inflammation during acute and early chronic SIVsab infection of pigtailed macaques (PTMs). Levels of proinflammatory cytokines were consistently lower in SIVsab-infected PTMs receiving RFX+SFZ (red) compared to untreated controls (black): TNF-α (a); I-TAC (b); and C-reactive protein (CRP) (c).
(PDF)

S5 Fig Rifaximin (RFX) and sulfasalazine (SFZ) improves the natural history of SIVsab infection during acute and early chronic SIVsab infection of pigtailed macaques (PTMs). Levels of viral replication were significantly lower in SIVsab-infected PTMs receiving RFX+SFZ (red) compared to untreated controls (black) (a). While no significant impact of RFX+SFZ treatment could be observed in circulating CD4^+^ T cells (b), a less prominent depletion of mucosal CD4^+^ T cells could be observed in treated PTMs (c).
(PDF)

S6 Fig Rifaximin (RFX) and sulfasalazine (SFZ) reduces the levels of coagulation biomarkers during acute and early chronic SIVsab infection of pigtailed macaques (PTMs). Levels of d-dimer (a) and tissue factor (b) were significantly lower in SIVsab-infected PTMs receiving RFX+SFZ (red) compared to untreated controls (black). Shown are the average values for each group and standard error of means.
(PDF)
