# Supplementary material for: Antibiotic and Antiinflammatory Therapy Transiently Reduces Inflammation and Hypercoagulation in Acutely SIV-Infected Pigtailed Macaques
Source: PLoS Pathog. 2016 Jan 14;12(1):e1005384. doi: 10.1371/journal.ppat.1005384 (PMC4713071; doi:10.1371/journal.ppat.1005384)
Supplement: S1 Fig — (PDF) [file ppat.1005384.s001.pdf]

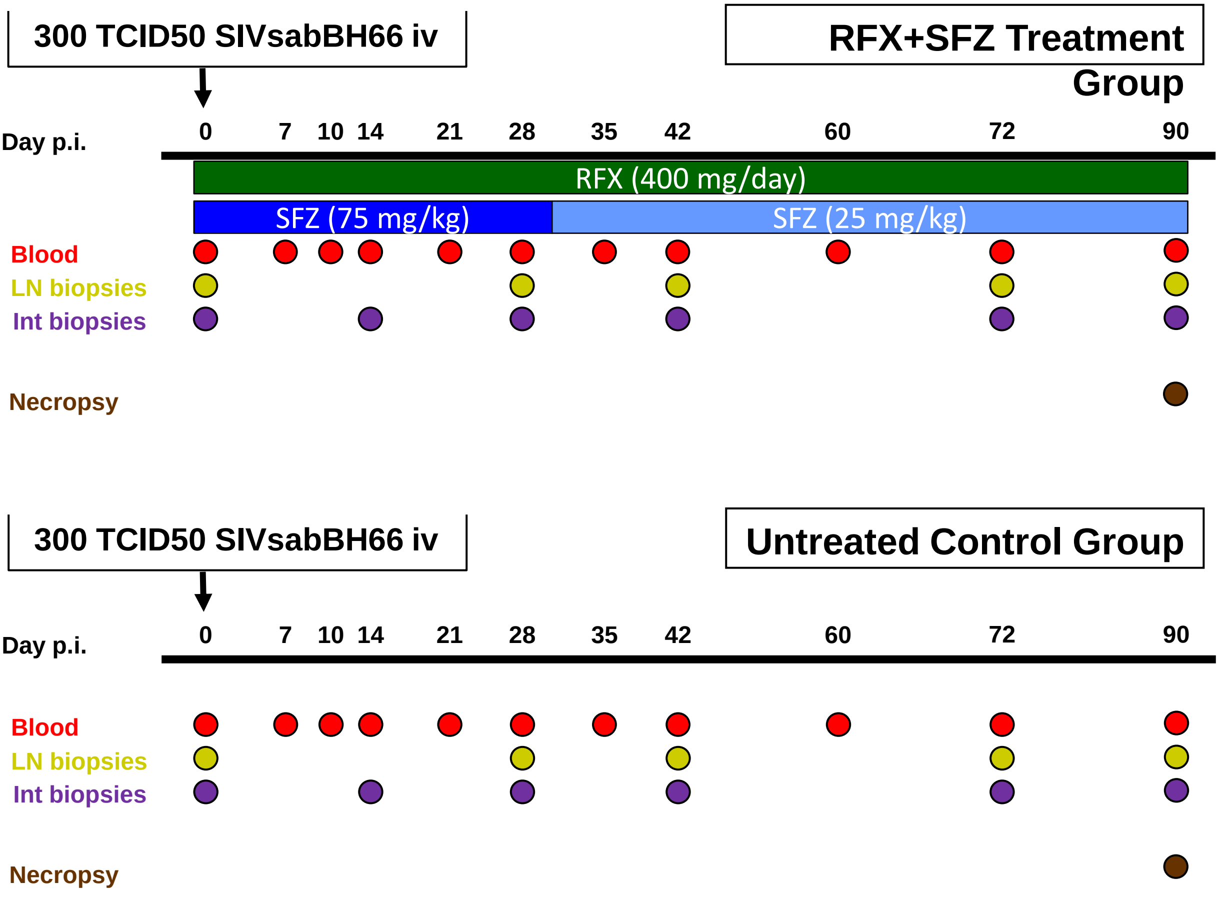

Figure S1. Treatment and sampling schedules for the PTMs receiving the RFX+SFZ treatment and for untreated controls.
