# Supplementary material for: Antibiotic and Antiinflammatory Therapy Transiently Reduces Inflammation and Hypercoagulation in Acutely SIV-Infected Pigtailed Macaques
Source: PLoS Pathog. 2016 Jan 14;12(1):e1005384. doi: 10.1371/journal.ppat.1005384 (PMC4713071; doi:10.1371/journal.ppat.1005384)
Supplement: S2 Fig — (a) Comparison between plasma LPS levels in SIVsab-infected PTMs receiving RFX+SFZ (red) and untreated controls (black). (b) Comparison between plasma sCD14 levels in SIVsab-infected PTMs receiving RFX+SFZ (red) and untreated controls (black). (PDF) [file ppat.1005384.s002.pdf]

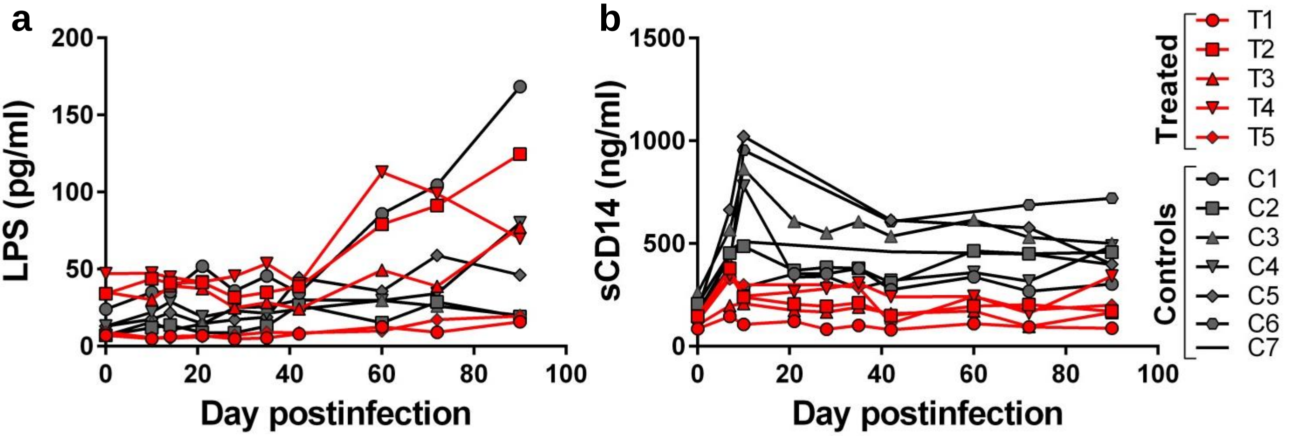

Figure S2. Rifaximin (RFX) and sulfasalazine (SFZ) treatment reduces microbial translocation during early SIVsab infection of pigtailed macaques (PTMs). (a) Comparison between plasma LPS levels in SIVsab-infected PTMs receiving RFX+SFZ (red) and untreated controls (black). (b) Comparison between plasma sCD14 levels in SIVsab-infected PTMs receiving RFX+SFZ (red) and untreated controls (black).
