# Supplementary material for: Antibiotic and Antiinflammatory Therapy Transiently Reduces Inflammation and Hypercoagulation in Acutely SIV-Infected Pigtailed Macaques
Source: PLoS Pathog. 2016 Jan 14;12(1):e1005384. doi: 10.1371/journal.ppat.1005384 (PMC4713071; doi:10.1371/journal.ppat.1005384)
Supplement: S3 Fig — Significant differences were observed between SIVsab-infected PTMs receiving RFX+SFZ (red) and untreated controls (black) with regard to CD38 and HLA-DR expression by CD4+ T cells (a) and CD8+ T cells (b). Conversely, Ki-67 expression by CD4+ T cells (c) and CD8+ T cells (d) was not statistically different between the two groups. (PDF) [file ppat.1005384.s003.pdf]

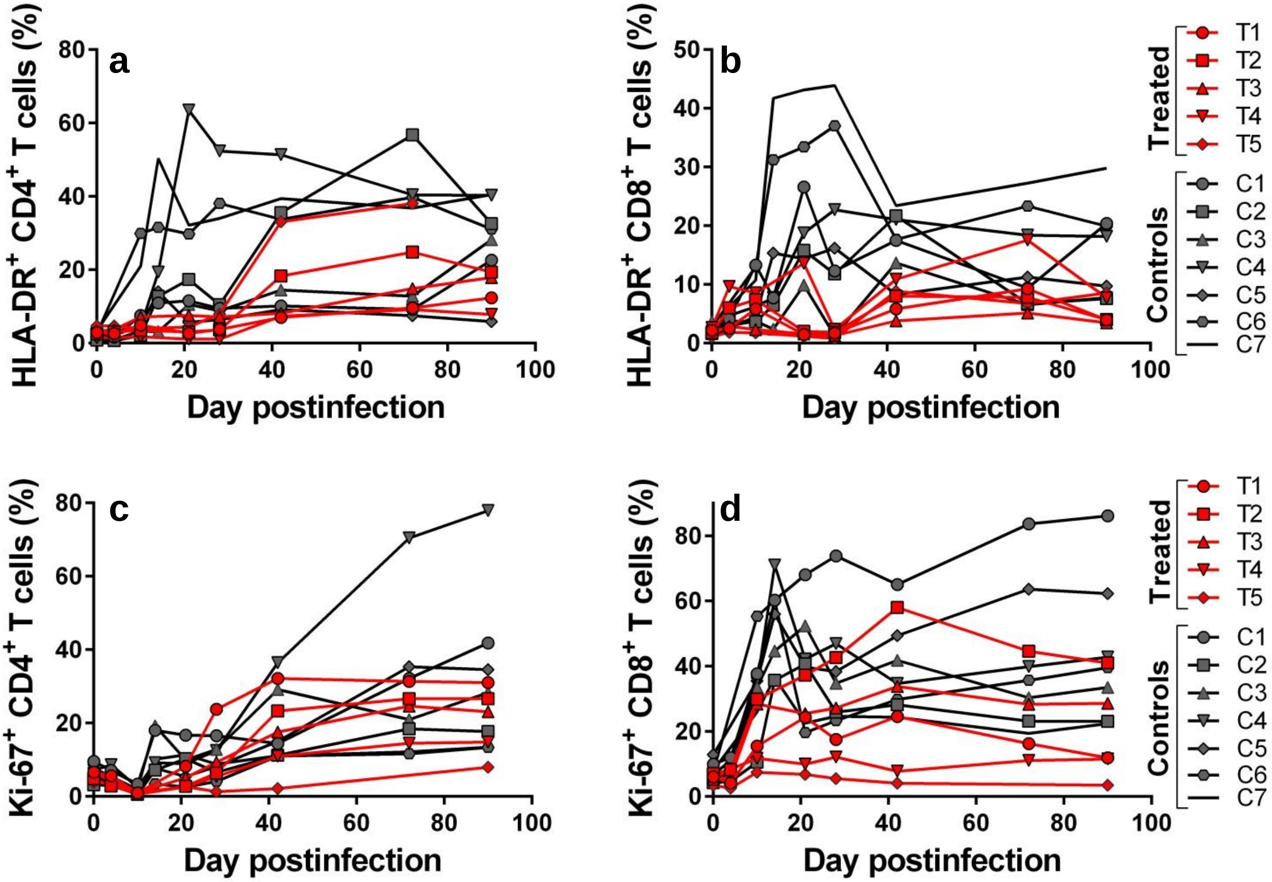

Figure S3. Rifaximin (RFX) and sulfasalazine (SFZ) treatment impacts T cell immune activation during acute and early chronic SIVsab infection of pigtailed macaques (PTMs). Significant differences were observed between SIVsab-infected PTMs receiving RFX+SFZ (red) and untreated controls (black) with regard to CD38 and HLA-DR expression by $CD4^+$ T cells (a) and $CD8^+$ T cells (b). Conversely, Ki-67 expression by $CD4^+$ T cells (c) and $CD8^+$ T cells (d) was not statistically different between the two groups.
