# Supplementary material for: Antibiotic and Antiinflammatory Therapy Transiently Reduces Inflammation and Hypercoagulation in Acutely SIV-Infected Pigtailed Macaques
Source: PLoS Pathog. 2016 Jan 14;12(1):e1005384. doi: 10.1371/journal.ppat.1005384 (PMC4713071; doi:10.1371/journal.ppat.1005384)
Supplement: S4 Fig — Levels of proinflammatory cytokines were consistently lower in SIVsab-infected PTMs receiving RFX+SFZ (red) compared to untreated controls (black): TNF-α (a); I-TAC (b); and C-reactive protein (CRP) (c). (PDF) [file ppat.1005384.s004.pdf]

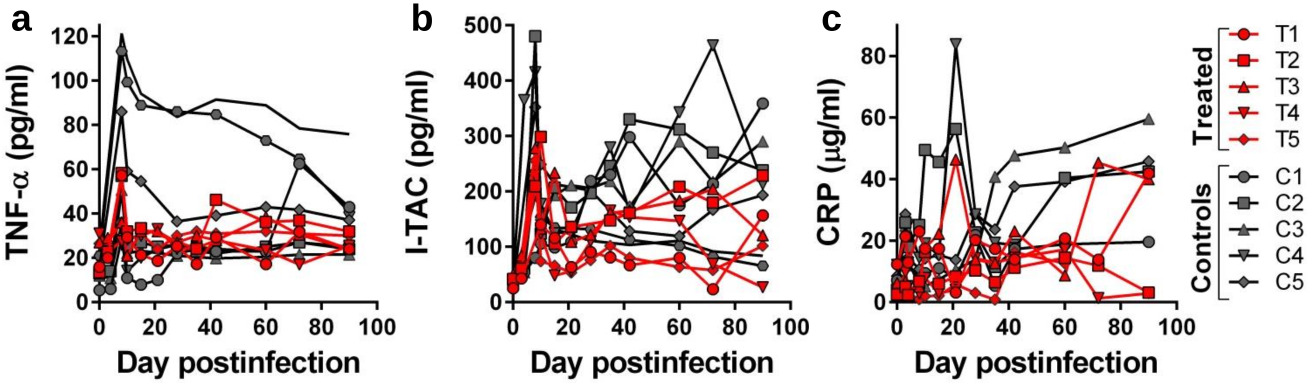

Figure S4. Rifaximin (RFX) and sulfasalazine (SFZ) impacts the levels of inflammation during acute and early chronic SIVsab infection of pigtailed macaques (PTMs). Levels of proinflammatory cytokines were consistently lower in SIVsab-infected PTMs receiving RFX+SFZ (red) compared to untreated controls (black): TNF-α (a); I-TAC (b); and C-reactive protein (CRP) (c).
