# Supplementary material for: Antibiotic and Antiinflammatory Therapy Transiently Reduces Inflammation and Hypercoagulation in Acutely SIV-Infected Pigtailed Macaques
Source: PLoS Pathog. 2016 Jan 14;12(1):e1005384. doi: 10.1371/journal.ppat.1005384 (PMC4713071; doi:10.1371/journal.ppat.1005384)
Supplement: S5 Fig — Levels of viral replication were significantly lower in SIVsab-infected PTMs receiving RFX+SFZ (red) compared to untreated controls (black) (a). While no significant impact of RFX+SFZ treatment could be observed in circulating CD4+ T cells (b), a less prominent depletion of mucosal CD4+ T cells could be observed in treated PTMs (c). (PDF) [file ppat.1005384.s005.pdf]

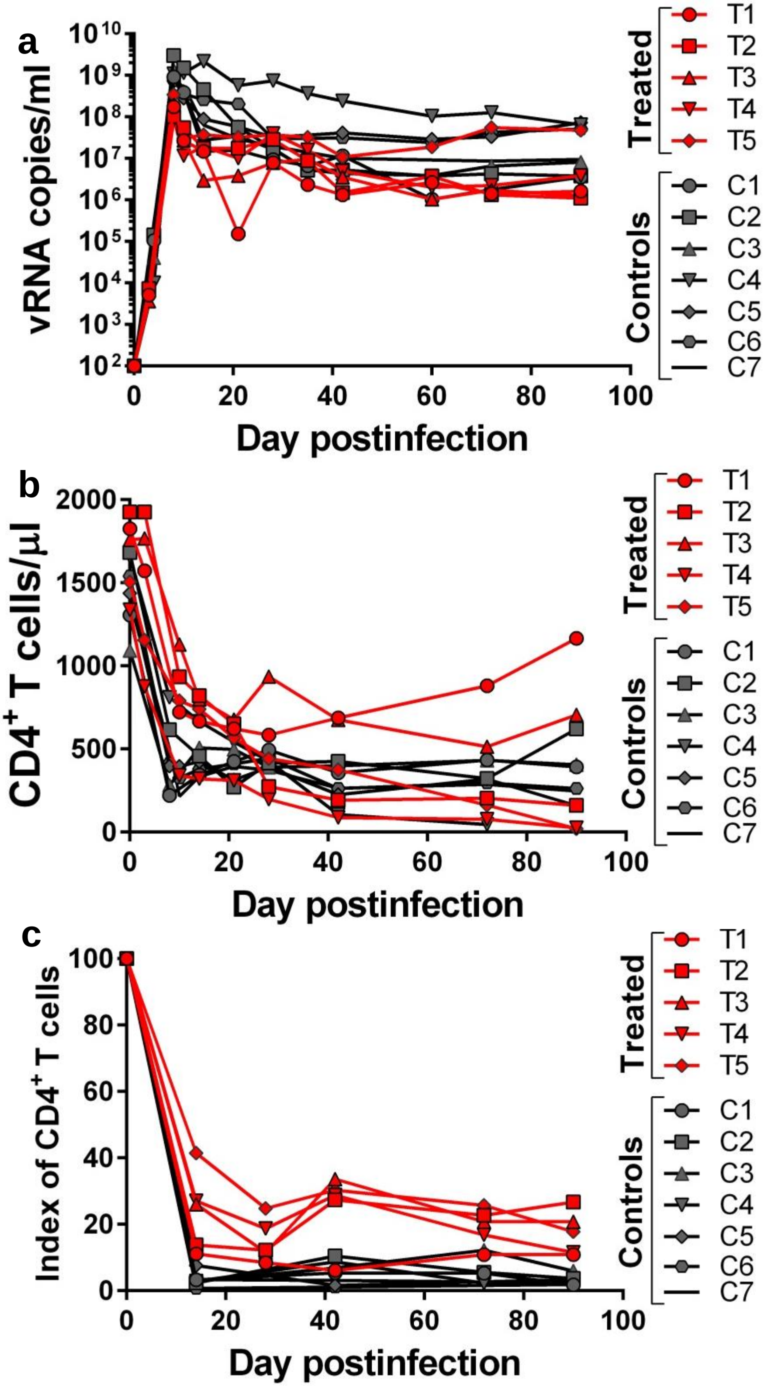

Figure S5. Rifaximin (RFX) and sulfasalazine (SFZ) improves the natural history of SIVsab infection during acute and early chronic SIVsab infection of pigtailed macaques (PTMs). Levels of viral replication were significantly lower in SIVsab-infected PTMs receiving RFX+SFZ (red) compared to untreated controls (black) (a). While no significant impact of RFX+SFZ treatment could be observed in circulating $CD4^+$ T cells (b), a less prominent depletion of mucosal $CD4^+$ T cells could be observed in treated PTMs (c).
