# Supplementary material for: Antibiotic and Antiinflammatory Therapy Transiently Reduces Inflammation and Hypercoagulation in Acutely SIV-Infected Pigtailed Macaques
Source: PLoS Pathog. 2016 Jan 14;12(1):e1005384. doi: 10.1371/journal.ppat.1005384 (PMC4713071; doi:10.1371/journal.ppat.1005384)
Supplement: S6 Fig — Levels of d-dimer (a) and tissue factor (b) were significantly lower in SIVsab-infected PTMs receiving RFX+SFZ (red) compared to untreated controls (black). Shown are the average values for each group and standard error of means. (PDF) [file ppat.1005384.s006.pdf]

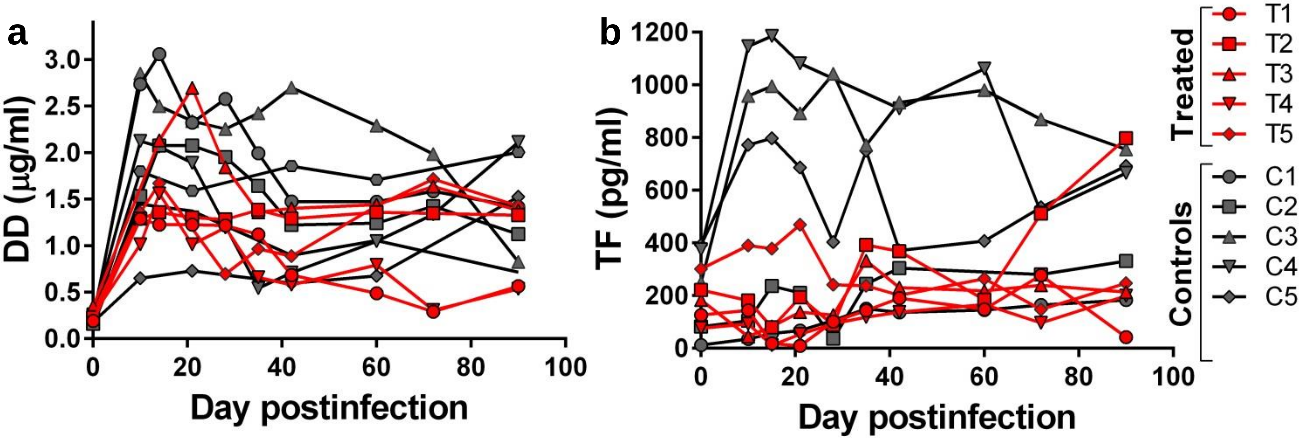

Figure S6. Rifaximin (RFX) and sulfasalazine (SFZ) reduces the levels of coagulation biomarkers during acute and early chronic SIVsab infection of pigtailed macaques (PTMs). Levels of d-dimer (a) and tissue factor (b) were significantly lower in SIVsab-infected PTMs receiving RFX+SFZ (red) compared to untreated controls (black). Shown are the average values for each group and standard error of means.
